# Gastric volvulus associated with preduodenal portal vein in right isomerism: a case report

**DOI:** 10.1093/jscr/rjag483

**Published:** 2026-06-22

**Authors:** Yen Nguyen Le, Tuyen Tran, Kiet Tuan Phan, Mai Ngoc Nguyen

**Affiliations:** Department of Pediatric Surgery, University of Medicine and Pharmacy at Ho Chi Minh City, Ho Chi Minh City, Vietnam; Department of Pediatrics, Vinmec Central Park International Hospital, Vinmec Healthcare System, Ho Chi Minh City, Vietnam; Children’s Hospital No. 2, Ho Chi Minh City, Vietnam; Children’s Hospital No. 2, Ho Chi Minh City, Vietnam

**Keywords:** preduodenal portal vein, gastric volvulus, right isomerism, heterotaxy syndrome, asplenia, case report

## Abstract

Preduodenal portal vein (PDPV) is a rare vascular anomaly associated with heterotaxy syndrome, typically presenting as duodenal obstruction in left isomerism. We report a 13-month-old male with right isomerism, single ventricle physiology, and asplenia who presented with postprandial vomiting. Computed tomography revealed a PDPV anterior to the pylorus, and upper gastrointestinal contrast study confirmed mesentericoaxial gastric volvulus. Surgery consisted of gastric derotation, ante-PDPV pyloroduodenostomy, and anterior gastropexy. The patient recovered uneventfully and remained asymptomatic at 9-month follow-up. To our knowledge, this is the first report of PDPV with right isomerism complicated by gastric volvulus. Absent gastric stabilizing ligaments in asplenia, combined with PDPV-related obstruction, may predispose to volvulus. Multimodal imaging and individualized surgical management are essential in this rare setting.

## Introduction

Preduodenal portal vein (PDPV) is a rare congenital anomaly in which the portal vein courses anterior to the duodenum, potentially causing gastrointestinal obstruction [1, 2]. In heterotaxy syndrome, PDPV has been predominantly reported in left isomerism presenting as duodenal obstruction [3, 4]. Right isomerism, characterized by asplenia and complex cardiac anomalies, predisposes to gastric volvulus through absent gastric stabilizing ligaments [5, 6]. The association of PDPV with right isomerism complicated by gastric volvulus has not been previously described. We present this novel combination, highlighting diagnostic and surgical considerations.

## Case report

A 13-month-old male (8.6 kg, 75 cm) with prenatally diagnosed single ventricle physiology was admitted with 2-day recurrent non-bilious vomiting. He had been under cardiac surveillance without prior intervention. Examination revealed mild abdominal distension with a palpable soft mass in the right hypochondrium. On admission he was unwell: febrile (39.5°C) with a heart rate of 144/min, respiratory rate 36/min, and oxygen saturation of 88%; blood pressure was not reliably recorded because of marked irritability, and capillary blood glucose was low at 41 mg/dl. He was resuscitated with isotonic saline (0.9% sodium chloride, 10 ml/kg/h), antipyretics, and a single intravenous bolus of 30% dextrose (2 ml/kg), with capillary blood glucose normalizing to 91 mg/dl within 2 h.

Abdominal ultrasonography identified a 105 × 50 mm cystic-appearing structure in the right flank, subsequently confirmed as the markedly dilated stomach. Following the abnormal ultrasound, surgical consultation was obtained and a nasogastric tube drained 200 ml of brown gastric fluid, confirming that gastric decompression was feasible. He remained febrile and tachycardic with oxygen saturation of 85%. Laboratory studies showed secondary polycythaemia (haemoglobin 15.5 g/dl, haematocrit 46.7%) consistent with chronic cyanosis; other values were white cell count 6.89 × 10^9^/L (48% neutrophils), platelets 390 × 10^9^/L, C-reactive protein 5.1 mg/L, AST/ALT 41/23 U/L, urea 14.4 mmol/L, creatinine 69 μmol/L, sodium 142 mmol/L, potassium 4.3 mmol/L, chloride 106 mmol/L, and troponin 0.0137 μg/L. Contrast-enhanced computed tomography (CT) demonstrated midline liver, absent spleen, markedly dilated right-sided stomach, and the portal vein coursing anterior to the pylorus and first duodenal portion (Fig. 1). Upper gastrointestinal contrast study (UGI) confirmed contrast retention with failure of duodenal passage and superior pyloric displacement suggesting mesentericoaxial volvulus (Fig. 2). Echocardiography confirmed right isomerism with single ventricle, complete atrioventricular septal defect, and severe pulmonary stenosis.

**Figure 1:**
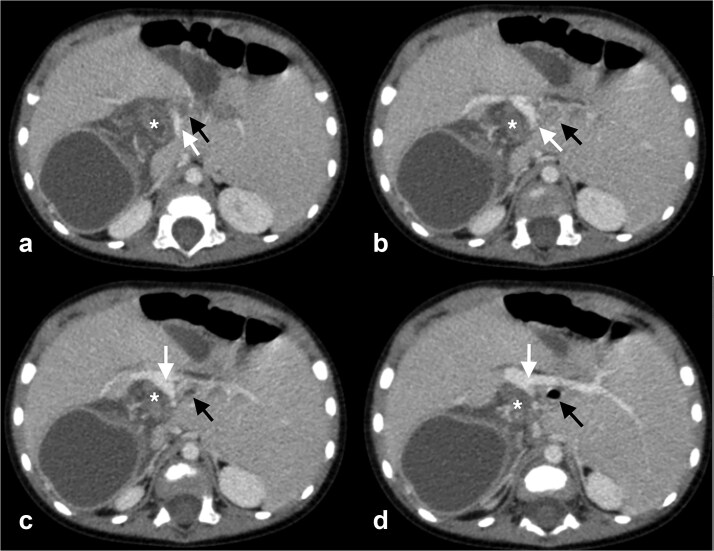
Contrast-enhanced axial CT images demonstrating PDPV anterior to the pylorus-duodenum. (a–d) Portal vein (white arrow) coursing anterior to the pylorus (asterisk) and duodenum (black arrow).

**Figure 2:**
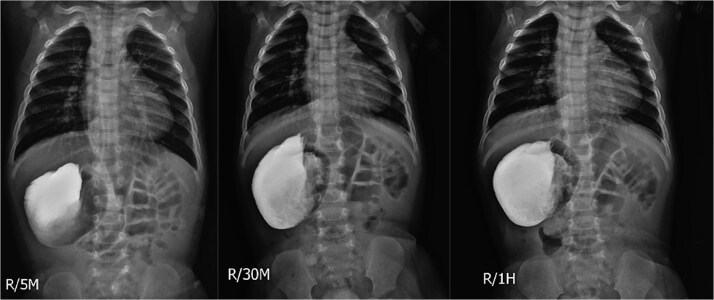
Serial upper GI contrast study at 5 minutes (left), 30 minutes (center), and 1 hour (right) showing right-sided stomach with progressive contrast retention and failure of duodenal passage. The pylorus is displaced superiorly with reduced pylorus-cardia distance, suggesting mesentericoaxial volvulus.

Given the severe cyanotic cardiac anomaly, a multidisciplinary cardiology and anaesthesia consultation was undertaken before surgery. The low oxygen saturation (85%–88%) and mildly detectable troponin were attributed to the underlying single-ventricle physiology rather than acute decompensation; no contraindication to surgery was identified, and an intraoperative re-consultation threshold of oxygen saturation <70% was set. Once fever and tachycardia had resolved with resuscitation and gastric decompression, the patient was deemed operable, and emergency surgery proceeded ~11–12 hours after admission.

Emergency laparoscopy revealed midline liver, absent spleen, ~270° mesentericoaxial gastric volvulus with mild cyanosis recovering after derotation, and PDPV crossing anterior to the pylorus–duodenum junction (Fig. 3). Intestinal malrotation was present. Conversion to open laparotomy was performed for precise dissection around the PDPV. The procedure comprised gastric derotation, transection and repositioning of the pylorus–duodenum anterior to the portal vein, ante-PDPV pyloroduodenostomy with interrupted 5–0 PDS sutures, anterior gastropexy, and jejunostomy tube placement (Fig. 4).

**Figure 3:**
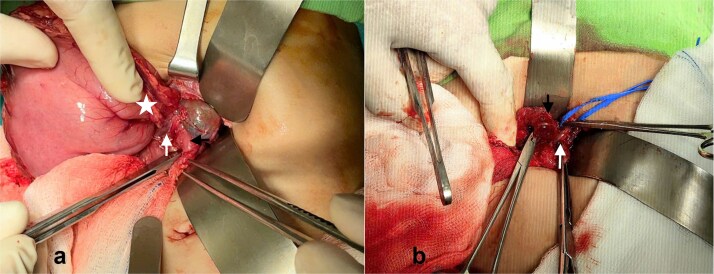
Intraoperative photographs. (a) PDPV (white arrow) compressing the pylorus (asterisk)-duodenum (black arrow) junction; (b) the pylorus–duodenum (black arrow) after transection and mobilization anterior to the portal vein (white arrow) in preparation for anastomosis.

**Figure 4:**
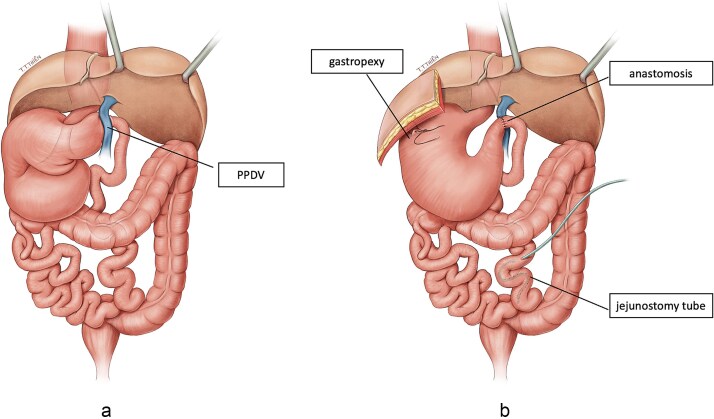
Schematic illustration of anatomical findings and surgical reconstruction in right isomerism with PDPV. (a) Preoperative anatomy showing midline liver, absent spleen, mesentericoaxial gastric volvulus, PDPV crossing anterior to the pylorus–duodenum junction, and intestinal malrotation. (b) Postoperative reconstruction following gastric derotation, ante-PDPV pyloroduodenostomy, anterior gastropexy, and jejunostomy tube placement.

The patient was extubated on postoperative day 1, tolerated full enteral feeds by Day 7, and was discharged on Day 10. At 6 weeks, one vomiting episode attributed to premature dietary advancement was confirmed not to represent anastomotic dysfunction by UGI (Fig. 5). At 9-month follow-up, the patient remained asymptomatic with normal growth.

**Figure 5:**
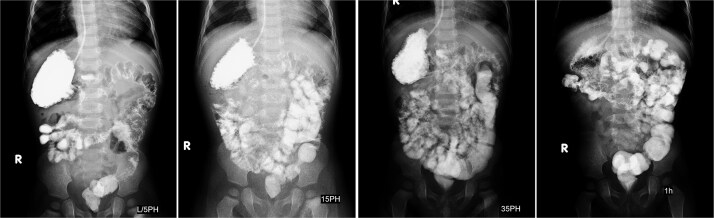
Follow-up upper GI contrast study at 6 weeks postoperatively (5 minutes, 15 minutes, 35 minutes, and 1 hour) demonstrating patent anastomosis with unobstructed contrast passage into the small bowel.

## Discussion

To our knowledge, this represents the first documented association of PDPV with right isomerism complicated by gastric volvulus. Previous reports have consistently linked symptomatic PDPV with left isomerism presenting as duodenal obstruction [3, 4, 7]. The distinct pathophysiology in our case warrants discussion.

Gastric volvulus in this patient resulted from multiple anatomical factors: asplenia eliminating the gastrosplenic and splenorenal ligaments that anchor the greater curvature; midline liver altering hepatogastric ligament orientation; and intestinal malrotation destabilizing gastric position [5, 8, 9]. PDPV may have contributed through partial gastric outlet obstruction and abnormal pyloric positioning, facilitating volvulus formation. Aoyama and Tateishi previously described gastric volvulus as a recognized complication of asplenia syndrome [10].

Multimodal imaging was essential for diagnosis. CT identified the PDPV, splenic absence, and liver position, while UGI was indispensable for demonstrating the volvulus pattern. Surgical management addressed both the mechanical obstruction and volvulus predisposition. The PDPV crossing at the pylorus–D1 junction made ante-PDPV pyloroduodenostomy feasible [11, 12], while concurrent gastropexy was essential given the underlying predisposition, consistent with recommendations for prophylactic gastropexy in asplenia [13]. In heterotaxy with complex cyanotic cardiac disease, a brief targeted resuscitation window with multidisciplinary cardiology and anaesthesia input allowed correction of an unstable acute presentation before emergency surgery, without delaying definitive treatment of the volvulus.

Clinically, surgeons should maintain a high index of suspicion for vascular anomalies in heterotaxy patients presenting with gastric volvulus, as failure to recognize PDPV may result in inadvertent vascular injury during surgical exploration. Limitations include the 9-month follow-up period and inherent constraints of single-case reports. Long-term cardiac morbidity may independently affect outcomes.

In conclusion, this case documents a novel association of PDPV with right isomerism and gastric volvulus. The combination of absent gastric stabilizing ligaments and PDPV-related obstruction creates a unique pathophysiological scenario requiring individualized surgical management combining ante-PDPV anastomosis with gastropexy.
